# Modeling and Analysis of a Direct Time-of-Flight Sensor Architecture for LiDAR Applications †

**DOI:** 10.3390/s19245464

**Published:** 2019-12-11

**Authors:** Preethi Padmanabhan, Chao Zhang, Edoardo Charbon

**Affiliations:** 1 AQUA Laboratory, École Polytechnique Fédérale de Lausanne (EPFL), 2000 Neuchâtel, Switzerland; edoardo.charbon@epfl.ch; 2 AQUA Laboratory, Delft University of Technology (TU Delft), 2628 CD Delft, The Netherlands; zhangchao.ac@hotmail.com

**Keywords:** direct time-of-flight (DTOF) sensor, flash LiDAR, background noise, wide dynamic range targets, coincidence detection, time-gating

## Abstract

Direct time-of-flight (DTOF) is a prominent depth sensing method in light detection and ranging (LiDAR) applications. Single-photon avalanche diode (SPAD) arrays integrated in DTOF sensors have demonstrated excellent ranging and 3D imaging capabilities, making them promising candidates for LiDARs. However, high background noise due to solar exposure limits their performance and degrades the signal-to-background noise ratio (SBR). Noise-filtering techniques based on coincidence detection and time-gating have been implemented to mitigate this challenge but 3D imaging of a wide dynamic range scene is an ongoing issue. In this paper, we propose a coincidence-based DTOF sensor architecture to address the aforementioned challenges. The architecture is analyzed using a probabilistic model and simulation. A flash LiDAR setup is simulated with typical operating conditions of a wide angle field-of-view (FOV = 40${}^{{}^{\circ}}$) in a 50 klux ambient light assumption. Single-point ranging simulations are obtained for distances up to 150 m using the DTOF model. An activity-dependent coincidence is proposed as a way to improve imaging of wide dynamic range targets. An example scene with targets ranging between 8–60% reflectivity is used to simulate the proposed method. The model predicts that a single threshold cannot yield an accurate reconstruction and a higher (lower) reflective target requires a higher (lower) coincidence threshold. Further, a pixel-clustering scheme is introduced, capable of providing multiple simultaneous timing information as a means to enhance throughput and reduce timing uncertainty. Example scenes are reconstructed to distinguish up to 4 distinct target peaks simulated with a resolution of 500 ps. Alternatively, a time-gating mode is simulated where in the DTOF sensor performs target-selective ranging. Simulation results show reconstruction of a 10% reflective target at 20 m in the presence of a retro-reflective equivalent with a 60% reflectivity at 5 m within the same FOV.

## 1. Introduction

Time-of-flight (TOF) has become a key depth-sensing technique in a wide range of light detection and ranging (LiDAR) applications spanning across consumer, automotive and industrial fields. Advancement in semiconductor miniaturization has led to compact solutions at smaller form factors, thus, making integrated TOF sensors almost natively available in mobile phones today. Automotive LiDAR, undoubtedly, is progressing at a rapid pace with growing interests in autonomous cars. With increasing range of applications, TOF sensors are only becoming increasingly relevant.

TOF sensors are basically categorized into their direct (DTOF) and indirect (ITOF) forms. ITOF sensors have been implemented in a number of consumer applications for ranging and depth mapping. Recent ITOF sensors have demonstrated high spatial resolution with increased ability to detect multiple objects over a large field-of-view (FOV) [1]. However, due to the fact that the illuminator modulation frequency in an ITOF system is directly proportional to the detection range while being inversely proportional to the achievable precision, they are limited to short-distance ranging (typically <30 m) [1,2,3]. A class of ITOF sensors based on short-pulse modulation and multi-tap lock-in pixels is becoming an attractive candidate due to higher achievable range resolution [4], however, it is currently limited to distances under 10 m [5]. Another drawback of ITOF sensors is their limited ability to distinguish two nearby objects [6]. DTOF sensors, on the other hand, are able to mitigate these challenges with detection ranges reaching up to several hundred meters [7,8], principally determined by the available optical power and their innate ability to discriminate multiple echoes easily [9].

This paper focuses on single-photon avalanche diode (SPAD) based DTOF sensors for LiDAR. DTOF image sensors based on time-correlated single-photon counting (TCSPC) are actively explored for their high speed and accurate ranging capabilities. Depth sensing in DTOF is achieved by transmitting a periodic light source (typically a pulsed laser) to a target and detecting the arrival of the reflected photons by high performance photodetectors such as avalanche photodiodes (APDs), SPADs or silicon photomultipliers (SiPMs). A supporting electronic circuitry then measures the arrival time of these photons. In DTOF sensors, time-to-digital converters (TDCs) are typically used for this purpose [8,10,11,12,13,14]. TDCs have been implemented at per-pixel level, reported in References [10,15], as well as in shared architectures [8,13]. Most often, due to the large volume of data being generated in a DTOF sensor and limited output bandwidth, shared architectures have become more viable solutions.

Depth sensing in LiDAR systems is a complex process due to a number of challenges. High background noise from ambient light is one of the primary challenges. LiDARs can be implemented in scanning or flash modes of operation. Scanning LiDARs usually benefit from an increased signal-to-background noise ratio (SBR) due to higher achievable optical power while scanning over only sections of the target FOV. However, the presence of moving mechanical parts in conventional scanning systems introduces long-term reliability concerns. Recent advancement in solid-state scanning LiDARS with MEMS-based mirrors are achieving smaller form factors with better design and fewer optical components. Flash LiDARs benefit from a much simpler system by illuminating the entire FOV simultaneously, the drawback however being lower achievable SBR at longer ranges. Recent developments in illumination and optics have offered innovative solutions through VCSEL (Vertical Cavity Surface-Emitting Laser) array technology and laser diode arrays which circumvent this issue to some extent.

At the sensor-level, ambient light suppression has been addressed by coincidence detection on chip [11,12,16], a well-known technique utilizing spatio-temporal closeness of photons within a laser pulse to filter out background noise photons. While TCSPC with coincidence detection have shown effective noise-filtering properties, imaging in a wide dynamic range scenario is an ongoing challenge. Other challenges include adverse weather conditions and coping up with scattering effects of fog/cloud in an outdoor environment.

The main contributions of this paper are as follows:

1. We will address the challenge of imaging in a high background noise scenario with particular emphasis on wide dynamic range targets based on coincidence detection and time-gating.
2. We propose a coincidence-based shared-DTOF sensor architecture to operate in a Flash LiDAR scenario. The architecture is analyzed using a probabilistic model simulated on MATLAB.
3. We outline a framework for the integrated implementation of the sensor based on simulations of the proposed architecture.

The paper is organized as follows; Section 2 provides a system-level analysis of a Flash LiDAR using a shared-DTOF sensing scheme. This will form the basis for Section 3 which describes the proposed architecture and its statistical model. The simulation results are presented and discussed in Section 4. Recommendations for future work and conclusions are drawn in Section 5.

## 2. Flash LiDAR with DTOF Sensors

### 2.1. Flash LiDAR Model

**Figure 1:**
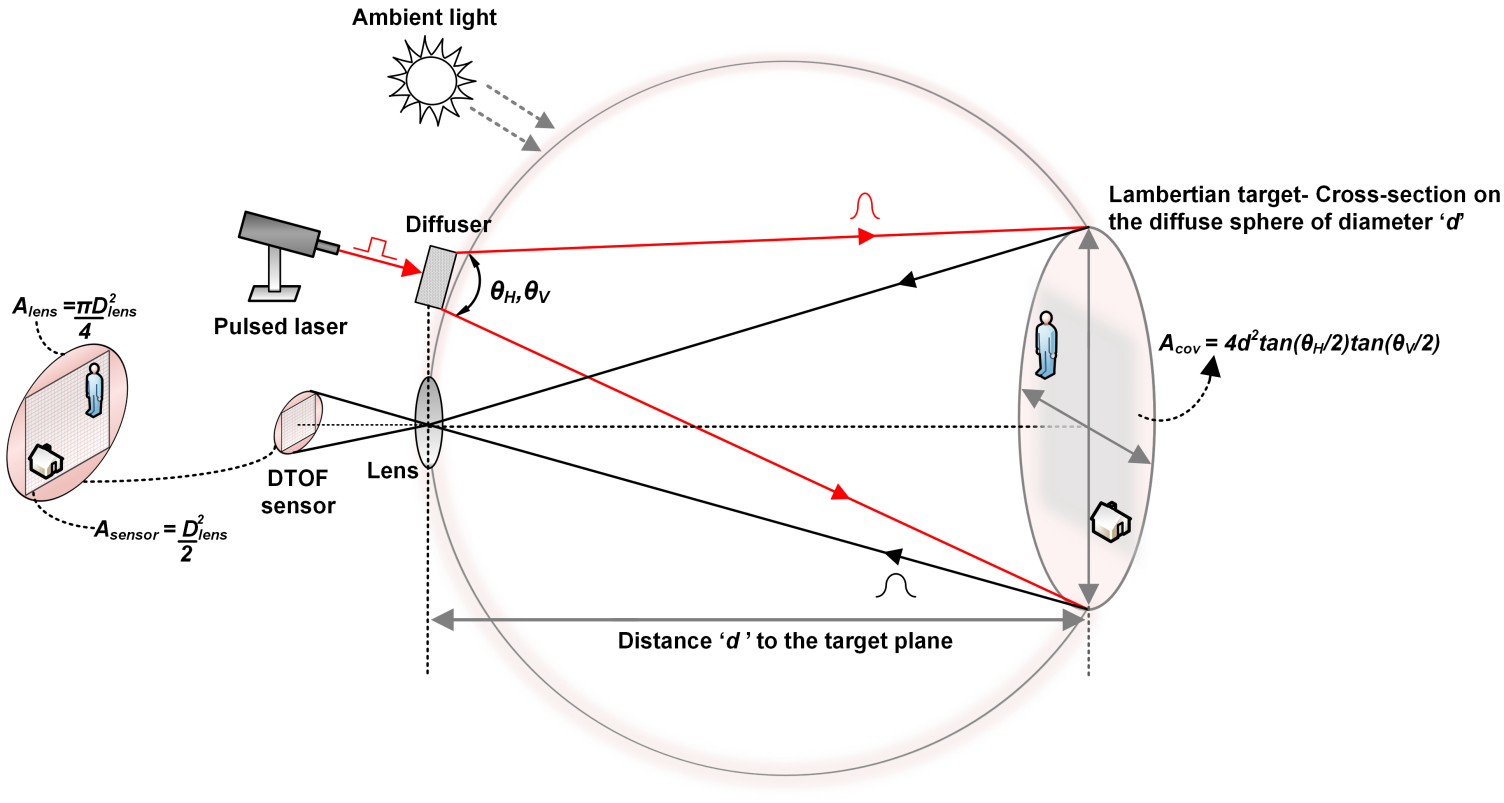
Flash LiDAR operation.

Figure 1 shows a generic flash LiDAR system where the scene of interest is uniformly illuminated using an active light source, typically, a wide-angle pulsed laser beam whose coverage area on a target plane depends on the fields-of-view (FOV) of the beam. A uniform illumination can be achieved by using an optical diffuser which spreads the incoming laser energy equally onto the coverage area or by illuminating the scene with a predefined light pattern, for instance, visualized as a matrix of light points covering the target area, where the number of those points may be defined based on the effective spatial resolution desired and the pixels in the LiDAR sensor itself. Irrespective of the method used for uniform illumination, the radiance of light on the target will comprise of both, the direct component and global component [17]. The direct component is basically the direct illumination of the laser source onto the target area which results in a single reflection onto the sensor while the global component may include indirect and/or multiple reflections caused from various physical phenomena. These could arise from inter-surface reflections simply due to the nature and geometry of different targets in the FOV or from light propagating through a scattering medium, such as, fog or cloud, resulting in ballistic and/or scattered photons.

Illuminating every point on a target scene using patterned light source allows us to mitigate the effects of global components, thus minimizing errors in range estimation and target recovery by primarily using only direct illumination [17,18]. However, it is important to mention that light transport involves interaction with targets made of diverse materials and geometry. While the direct component is easier to model, the global component, due to its aforementioned nature is complicated to model. Prior work show many efforts in the direction of modeling and separating direct and global components of light [17,18,19]. It is not in scope of this paper to analyze this further and therefore, only direct illumination from Lambertian targets has been considered throughout the paper. Furthermore, while the physical phenomena related to scattering medium such as fog or cloud will not be discussed, a time-gating feature in the proposed architecture will be discussed in Section 4.4, as a possible way to detect photons is such scenarios, particularly exploiting the advantages in a DTOF sensing method.

As shown in Figure 1, a pulsed laser with wavelength, $\lambda_{laser}$ and repetition rate, $f_{laser}$, is uniformly illuminated over a target scene at distance, *d*. The target is assumed to be Lambertian in nature, thus, reflecting photons over a diffuse sphere with diameter, *d* [20,21]. The horizontal and vertical FOV, $\theta_{H}$ and $\theta_{V}$ respectively, on the target plane are obtained by applying Pythagoras theorem and the corresponding coverage area, $A_{cov}$, over the scene is then written as,

(1) $$A_{cov}=4d^{2}\cdot tan\left(\frac{\theta_{H}}{2}\right)\cdot tan\left(\frac{\theta_{V}}{2}\right).$$

**Figure 2:**
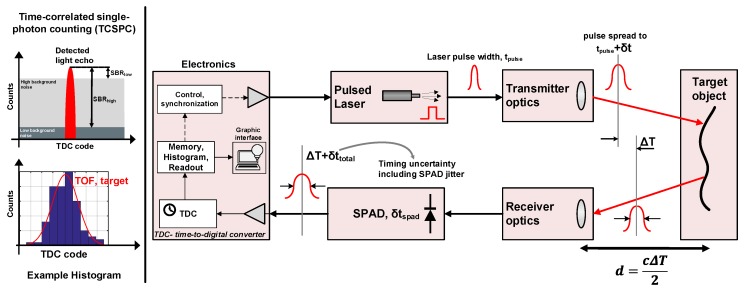
Principle of a direct time-of-flight (DTOF) sensor.

The photons back-reflected from the target are collected through a receiving lens with a diameter, $D_{lens}$, assuming that the light propagation path does not observe any scattering after which they are detected by the sensor. Due to the fact that the entire scene is illuminated at once, a sensor with an array of pixels is desired in order to reconstruct the image covered by the FOV [13,14]. A SPAD-based DTOF sensor has been considered for all analyses in this paper. The block diagram showing the principle of DTOF sensing is shown in Figure 2. The returning photons are detected by the SPAD and a time-stamping circuitry, typically a time-to-digital converter (TDC), measures the time of arrival of these photons with a certain resolution, $TDC_{res}$.

A DTOF measurement is generally performed using TCSPC, where detected events are accumulated over multiple laser pulses shone onto the target. The recovered signal is a train of pulses represented as a histogram corresponding to the time-of-arrival of individual photons incident on the SPAD with a distinguishable peak centered around the target location [8,10,11]. The measured timing information (ΔT in Figure 2) is then combined with the speed of light, *c*, to estimate the distance, *d*, of the target from the sensor.

While the TDC timestamps the detected photons with a given uncertainty determined by its root-mean-square (RMS) quantization error ($\sigma_{TDC}=TDC_{res}/\sqrt{12}$), the computed histogram is however, representative of all the other sources of timing uncertainties (shown as offsets or delays, $\Delta T+\delta t_{total}$ in Figure 2) arising from the laser pulse, the SPAD and any electrical circuit through the propagation of the detected event. Thus, the RMS of this total timing uncertainty, $\sigma_{total}$, is then given by the summation of the RMS values of the individual contributors assuming that they are all statistically independent.

(2) $$\sigma_{total}=\sqrt{\sigma_{laser}^{2}+\sigma_{SPAD}^{2}+\sigma_{TDC}^{2}+\sigma_{other}^{2}},$$

where the component, $\sigma_{other}$ accounts for jitter from any additional electronic circuitry through the propagation of the photon-event such as a combination tree which may combine events from multiple pixels and the surrounding logic, although, the contribution of this component can be considered negligible compared to other sources, such as the SPAD jitter (assumed to be on the order of 100 ps FWHM).

In an ideal scenario of low-noise condition, the target peak can be readily identified in the computed histogram, as shown conceptually in Figure 2 with $SBR_{high}$. However, most LiDAR applications suffer from high ambient light, in addition to the inherent detector noise itself (referred to as dark counts), thereby, significantly increasing the noise floor ($SBR_{low}$ in Figure 2). Most often, ambient light is the dominant source of noise, being several orders of magnitude (at least 2–3 orders of magnitude) higher than the detector dark count rate (DCR). Therefore, only ambient light will be considered for analysis in this paper. Also, the afterpulsing phenomenon is assumed negligible and therefore, not analyzed for simulations in this paper.

In addition to the signal photons from the laser, the sensor under solar exposure is subjected to the direct light coming from the sun as well as the back-reflected solar photons from the target and from the environment. The sensor collects light through the receiving lens irrespective of whether it is a laser or a solar photon. While the dominant effect is given by the solar photons back-reflected from the target FOV, there may still be direct sunlight photons hitting the surface of the sensor, contributing to the background noise floor in the optical path between the sensor and the collecting lens, although negligible in comparison to the solar photons integrated over the entire FOV on the target. The light illuminance in a typical indoor environment may be on the order of 5–10 klux while reaching up to 50–100 klux under a direct sunlight exposure (≈1050 W/m${}^{2}$) in an outdoor scenario [22]. A high background noise condition, as this can easily saturate the sensor, paralyzing its ability to detect the returning signal from the target itself [11,14]. Optical bandpass filters centered around the laser wavelength are commonly used to filter out background noise photons but they also have limited performance under high ambient light.

Assuming a flash scenario as seen in Figure 1, one can estimate the effective SBR from the number of noise events versus the number of signal events on a per-pixel basis. Background noise is modeled with Planck’s law of blackbody radiation and Poisson statistics; with an exposure to a solar irradiance, $P_{solar}$ W/m${}^{2}$, the returning power per pixel (in units of Watts) back-reflected from a flat target with uniform reflectivity, *r*, received through the lens with an efficiency, $T_{l}$ and filtered using an optical bandpass filter with a passband wavelength, $\Delta_{bw}$ and efficiency, $T_{f}$ can be written as follows

(3) $$P_{noise,pixel}=P_{solar}\times A_{cov}\times r\times {\left(\frac{D_{lens}}{2d}\right)}^{2}\times T_{l}\times T_{f}\times \Delta_{bw}\times \left(\frac{2}{\pi }\right)\times \left(\frac{1}{N}\right),$$

where, *N* is the number of pixels in the sensor. Due to the rectangular geometry of the sensor array, the entire area projected by lens which is circular, is not entirely useful. The effective area is accounted for, by multiplying the above equation by the fraction, $A_{sensor}/A_{lens}=2/\pi$, as shown in Figure 1. On expanding the term, $A_{cov}$ according to Equation (1), it can be observed that $P_{noise,pixel}$ is independent of the distance to the target, *d*. The reflected power per pixel from the laser pulse, with an average power, $P_{avg}$ can be similarly estimated as follows

(4) $$P_{signal,pixel}=P_{avg}\times r\times {\left(\frac{D_{lens}}{2d}\right)}^{2}\times T_{l}\times T_{f}\times \Delta_{bw}\times \left(\frac{2}{\pi }\right)\times \left(\frac{1}{N}\right).$$

Unlike noise, the returning power of the signal is dependent on the distance, *d* and decreases with $d^{2}$, following the inverse square law. Given a certain photon detection probability (PDP) for the SPAD and fill-factor, FF, will reduce the Equations (3) and (4) to

(5) $$P\_eff_{noise,pixel}=P_{noise,pixel}\times PDP\times FF,$$

(6) $$P\_eff_{signal,pixel}=P_{signal,pixel}\times PDP\times FF.$$

From the wavelength, $\lambda_{laser}$, of the laser, one can estimate the number of noise and signal events per second by dividing Equations (5) and (6) by the energy of the photon at $\lambda_{laser}$,

(7) $$N_{pixel}=\frac{P\_eff_{noise,pixel}}{hc/\lambda_{laser}},$$

(8) $$S_{pixel}=\frac{P\_eff_{signal,pixel}}{hc/\lambda_{laser}},$$

where *c* is the speed of light ($3\times 10^{8}$ ms${}^{-1}$) and *h*, Plancks’s constant ($6.628\times 10^{-34}$ Js).

SPADs have a certain dead time between successive detections which limits the theoretically estimated photon-count statistics. This dead time itself can be paralyzable or non-paralyzable in nature [23]. A non-paralyzable dead time, $t_{d}$, is assumed for all analyses in the paper. Therefore, the event rates in Equations (7) and (8) are modified to provide the effective rates.

(9) $$N\_eff_{pixel}=\frac{N_{pixel}}{1+N_{pixel}\cdot t_{d}},$$

(10) $$S\_eff_{pixel}=\frac{S_{pixel}}{1+S_{pixel}\cdot t_{d}}.$$

For simplicity, we will continue using the terms, $N_{pixel}$ and $S_{pixel}$, for the noise and signal event rates respectively. Based on the established equations, the effective noise and signal events per pixel per second were simulated for a flat target of r=10% reflectivity over varying distances. An average laser power, $P_{avg}$, of 20 mW, wavelength, $\lambda_{laser}=780$ nm and a repetition rate of 1 MHz and a pulsewidth of ≈500 ps were assumed to be uniformly illuminating the target with FOVs, $\theta_{H}=20^{{}^{\circ}}$ and $\theta_{V}=20^{{}^{\circ}}$. A 780 nm laser wavelength was used for analysis purposes simply because of the practical availability of such a laser in view of future measurements using that laser. However, a common choice for LiDAR is working with longer near-IR wavelengths, which would naturally perform better under high solar exposure compared to a 780 nm laser. A lens with $D_{lens}=11$ mm and a f-number of 1.4 (focal length ≈ 15 mm) was assumed to collect light onto a 32 × 32 SPAD sensor (N=1024). Additionally, background light of different levels from 5 klux (≈50 W/m${}^{2}$) to 100 klux (≈1000 W/m${}^{2}$) solar exposure was imposed without changing the laser parameters. A summary of common simulation parameters used through the paper is mentioned in Table 1.

**Figure 3:**
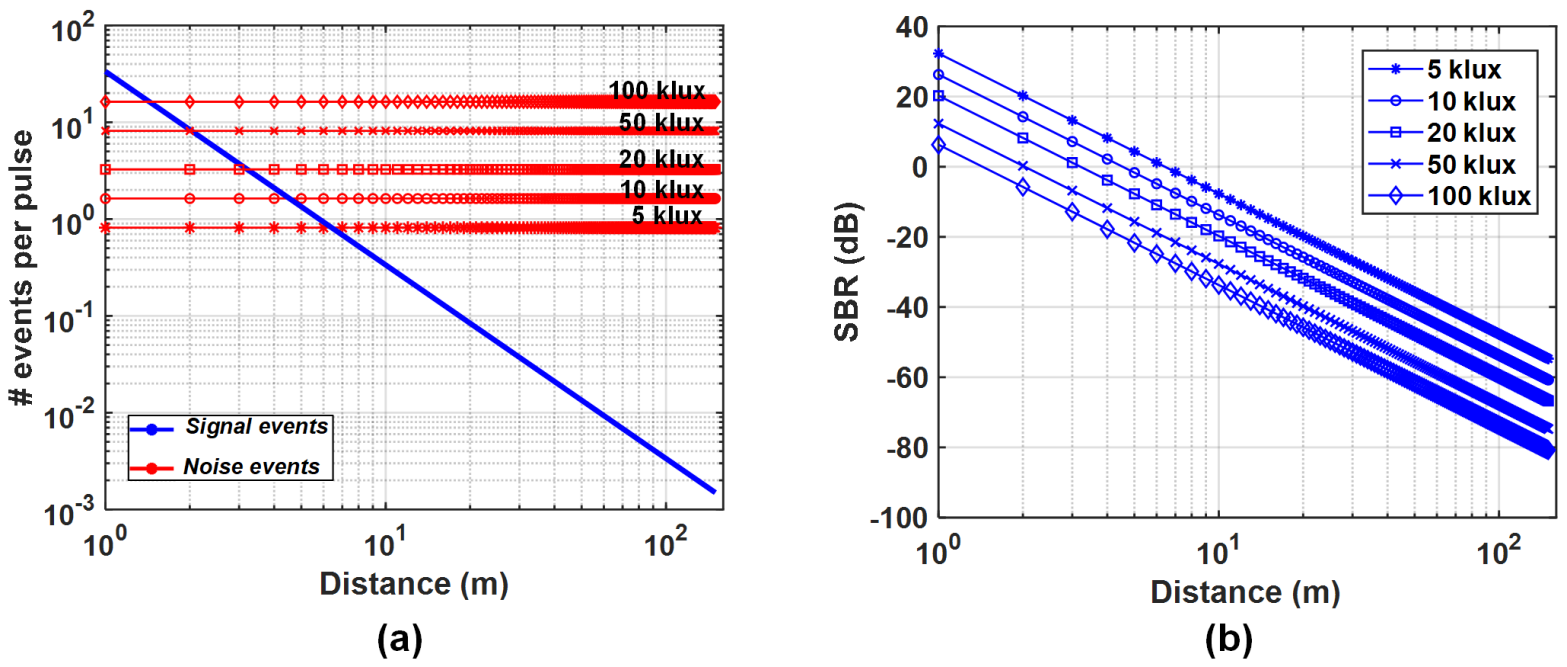
Simulation results of (**a**) the number of events per pixel per laser pulse at different background noise levels and (**b**) the SBR for 1–150 m target distances, *d*.

Figure 3 shows the resulting noise and signal rates indicated per laser pulse per pixel; no particular noise filtering mechanism has been modeled for this simulation. As can be seen in Figure 3b, beyond 1 m at 100 klux background light and beyond 2 m at 50 klux background light (≈503 W/m${}^{2}$), the system starts approaching a negative SBR regime, exemplifying the requirement to have effective noise filtering.

### 2.2. Analytical Model of a DTOF Sensor

**Figure 4:**
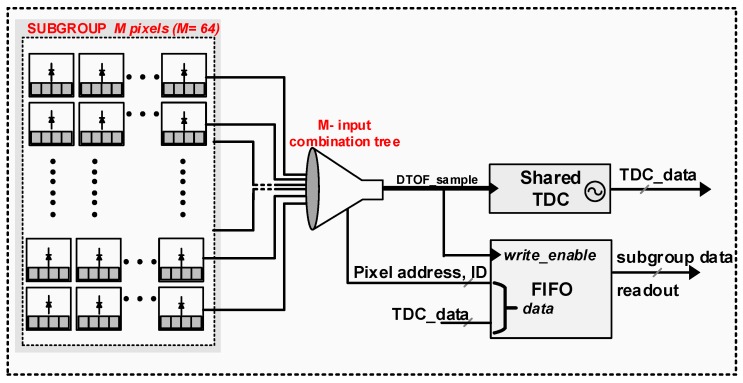
A block diagram of a DTOF sensor in a shared architecture.

With a known sensor architecture, the photon-detection process can be modeled analytically and the probability of signal detection can be calculated from noise and signal events obtained from Equations (7) and (8). For the analyses in this paper, a modular DTOF sensor in a shared architecture will be considered. The spatial arrangement of the pixels is inspired from one of our previous works [8]. An example sensor resolution of 32 × 32 SPAD pixels is considered throughout the paper with no particular limitation of scaling to larger formats. The sensor is visualized as an array of modules, called, subgroups, where every subgroup is clustered into an array of M= 8 × 8 SPADs. Therefore, it suffices to understand the operation of the sensor through a subgroup itself. Figure 4 shows a high-level block diagram of the subgroup within a shared TDC architecture.

Two such subgroups share an always-on TDC where the subgroup size has been assumed based on the achievable activity rate for a given incoming photon flux and power efficiency [24]. Within every subgroup is a combination tree which propagates the incoming events down to the TDC based on a first-in-win-all scheme, thus providing the timestamp of every first event [8]. The combination logic circuit has an associated dead-time, $t_{d,comb}$, after which it resets itself making it available for successive events. In a shared architecture as this, the total dead time, $t_{d}$ for detection is a combination of $t_{d,comb}$ and the SPAD dead time, $t_{d,spad}$. However, the overall SPAD dead time in a shared case reduces to ≈$t_{d,spad}/M$, thus making $t_{d,comb}$ the dominant contributor. Consequently, the maximum TDC conversion rate in a shared architecture as this is given by the inverse of the tree dead time, ≈$1/t_{d,comb}$, which can reach up to Gtimestamps/s [24].

Within the subgroup is also an address decoding logic which provides the “ID” of the pixel which detected the “first event”. The ID information along with the associated timestamp (TDC code) is then read out using a digital readout logic, such as a first-in-first-out (FIFO) bus. With this information on the sensor architecture, it is possible to estimate the probability of signal detection analytically for a Flash scenario described in Section 2.1. From the count statistics per pixel in Equations (7) and (8), the number of signal events with one laser pulse for every pixel can be calculated as

(11) $$N_{pulse,pixel}\left(i\right)=N_{pixel}\left(i\right)\times t_{meas},$$

(12) $$S_{pulse,pixel}\left(i\right)=S_{pixel}\left(i\right)\times \left(1/f_{laser}\right).$$

While the number of signal photons are directly related to the repetition rate of the laser (lower the repetition rate, higher is the energy per laser pulse), in that, all the laser photons are concentrated within the pulsewidth of the laser (FWHM) over the laser period (duty cycle ratio = $FWHM\times f_{laser}$), the number of integrated noise photons, however, need to be calculated based on the actual measurable window, $t_{meas}$, as shown in Equation (11).

The detection of incoming photon events is modeled as a Poisson arrival process with the probability of detecting *k* number of events over a measurable window, *t*, given by

(13) $$\begin{array}{c} p\left(k\right)=\frac{{\left(\lambda \left(t-kt_{d,spad}\right)\right)}^{k}\exp\left(-\lambda \left(t-kt_{d,spad}\right)\right)}{k!};t_{k}<t-t_{d,spad}, \end{array}$$

(14) $$\begin{array}{c} p\left(k\right)=\frac{{\left(\lambda \left(t_{k}-\left(k-1\right)t_{d,spad}\right)\right)}^{k}\exp\left(-\lambda \left(t_{k}-\left(k-1\right)t_{d,spad}\right)\right)}{k!};t_{k}>t-t_{d,spad}, \end{array}$$

where λ is the average photon arrival rate (not to be confused with $\lambda_{laser}$, the wavelength of the laser) and $t_{k}$ is the arrival time of these photons. Equation (14) is for the case when $kt_{d,spad}$ may fall outside the measurable window, *t*, arriving at a time $t_{k}$. For the given sensor architecture, the subgroup detects and propagates every first event (k=1) in an array of *M* pixels. A constant dead time of $t_{d,comb}$ is assumed during which the subgroup cannot detect any event and at the end of this duration, it is assumed that it is ready to detect the next incoming event. Thus, any detection within the subgroup following this time instant will now include the absolute dead time of the SPAD, $t_{d,spad}$. Under these conditions, Equations (11) and (12) are now modified to include the dead time of the SPAD as follows

(15) $$N_{pulse,pixel}\left(i\right)=N_{pixel}\left(i\right)\times \left(t_{meas}-t_{d,spad}\right),$$

(16) $$S_{pulse,pixel}\left(i\right)=S_{pixel}\left(i\right)\times \left(1/f_{laser}-t_{d,spad}\right).$$

The probability of detecting a noise event per pixel within a subgroup will be determined by the probability that the combination tree propagates this event through the subgroup. For every pixel, *i* in the subgroup, this will be given by the conditional probability of detecting a noise photon in pixel, *i*, given that no other noise event has been propagated through rest of the subgroup. In order to calculate this, the total number of photons per pulse within the subgroup, sg, is first estimated. The number of photons in a subgroup, sg, with *M* number of pixels, is calculated as a summation of the pixel-wise photon number for noise and signal per laser pulse.

(17) $$N_{pulse,sg}=\sum_{i=1}^{i=M}N_{pulse,pixel}\left(i\right),$$

(18) $$S_{pulse,sg}=\sum_{i=1}^{i=M}S_{pulse,pixel}\left(i\right).$$

The probability of detecting a noise event in pixel, *i* is given by

(19) $$p_{n,pixel}\left(i\right)=N_{pulse,pixel}\left(i\right)\exp\left(-N_{pulse,pixel}\left(i\right)\right).$$

The probability of detecting a noise event in subgroup sg in rest of the M−1 pixels excluding the $i^{th}$ pixel is given by

(20) $$p_{n,sg}\left(i\right)=\left(N_{pulse,sg}-N_{pulse,pixel}\left(i\right)\right)\exp\left(N_{pulse,sg}-N_{pulse,pixel}\left(i\right)\right).$$

The conditional probability is then calculated which provides the final effective probability of detecting and propagating a noise event through the subgroup.

(21) $$p_{n,eff}\left(i\right)=p_{n,pixel}\left(i\right)\times \left(1-p_{n,sg}\left(i\right)\right).$$

For the signal events, the final conditional probability can be calculated as follows

(22) $$p_{s,eff}\left(i\right)=\left(1-p_{n,pixel}\left(i\right)\right)\times \left(1-p_{n,sg}\left(i\right)\right)\times p_{s,pixel}\left(i\right),$$

where $p_{s,pixel}\left(i\right)$ is,

(23) $$p_{s,pixel}\left(i\right)=S_{pulse,pixel}\left(i\right)\exp\left(-S_{pulse,pixel}\left(i\right)\right).$$

**Figure 5:**
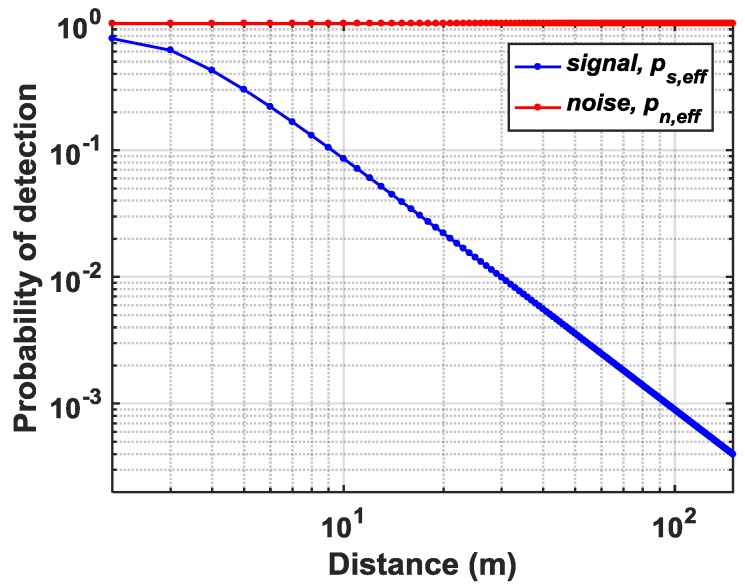
Probability of detecting signal and noise events in a Flash scenario using first-in-win-all DTOF scheme.

For the Flash LiDAR scenario described in Section 2.1, the probabilities of noise ($p_{n,eff}$) and signal ($p_{s,eff}$) detections are calculated for k=1, where the subgroup propagates every incoming first event. The simulated results from MATLAB are shown in Figure 5. All other simulation parameters remain the same except FOVs which are now increased to $\theta_{H}=40^{{}^{\circ}}$ and $\theta_{V}=40^{{}^{\circ}}$. The integration window for noise events is $t_{meas}=20$ ns. For most analysis in this paper, a 50 klux background light condition has been assumed unless specified otherwise.

As can be seen, due to the first-in-win-all nature of the subgroup and no particular noise-filtering mechanism, all the noise events are integrated over all pixels being illuminated during the entire measurement window, $t_{meas}$, therefore, making it practically impossible to detect signal events. Also, in a flash LiDAR which integrates background noise over a wide FOV (in this example, $40^{{}^{\circ}}$), the optical bandpass filter becomes ineffective at a 50 klux ambient light condition. Thus, it is paramount that the sensor architecture offers noise-filtering apart from optical methods, especially when high background noise as this example is of major concern.

## 3. Proposed DTOF Sensor Model

### 3.1. DTOF Sensor Adapted for Coincidence Detection

**Figure 6:**
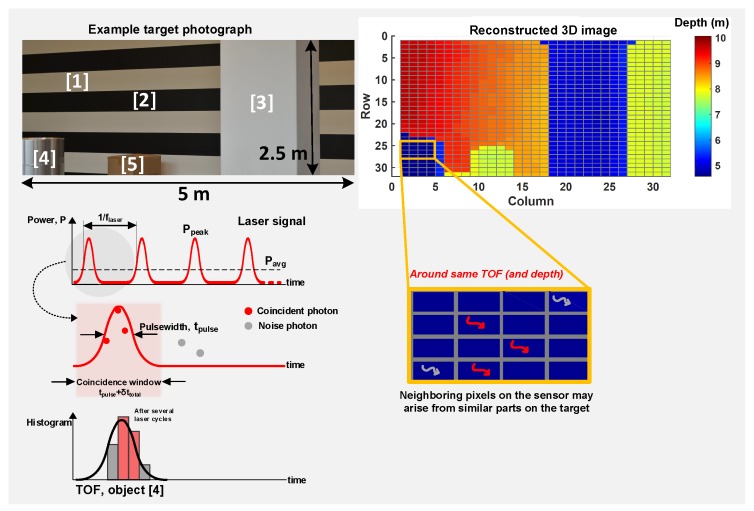
Conceptual representation of coincidence detection; the depth reconstruction is a measured image from Reference [8], utilized for analysis purposes.

Mitigating challenges from high background noise have been addressed by coincidence detection (or similar technique) on the sensor [7,11,12,16]. Coincidence detection is a well-known technique which utilizes spatio-temporal correlation of photons within a laser pulse to filter out background noise photons which are uniformly distributed in time. Figure 6 conceptually explains this technique with an example scene and a measured 3D image reconstruction [8].

The main idea is to exploit the fact that the signal photons reflected from the target are temporally correlated and thus, most likely to be concentrated within a time-window coarsely equal to the total system full width at half maximum, $FWHM\approx 2.355\sigma_{total}$, where $\sigma_{total}$ is as defined in Equation (2). Instead of letting the sensor integrate events over the entire measurement window, $t_{meas}$, imposing this time constraint, referred to as the “coincidence window”, reduces the likelihood of acquiring noise events whose probability of occurence within that window is very low, thus, electrically enhancing the SBR. Coincidence may be implemented at the sensor level over clusters/groups of closely-spaced pixels, exploiting a “more-likely” fact that neighboring pixels may belong to similar target depths (and thus, TOFs), as depicted in Figure 6 for the object labelled [4].

**Figure 7:**
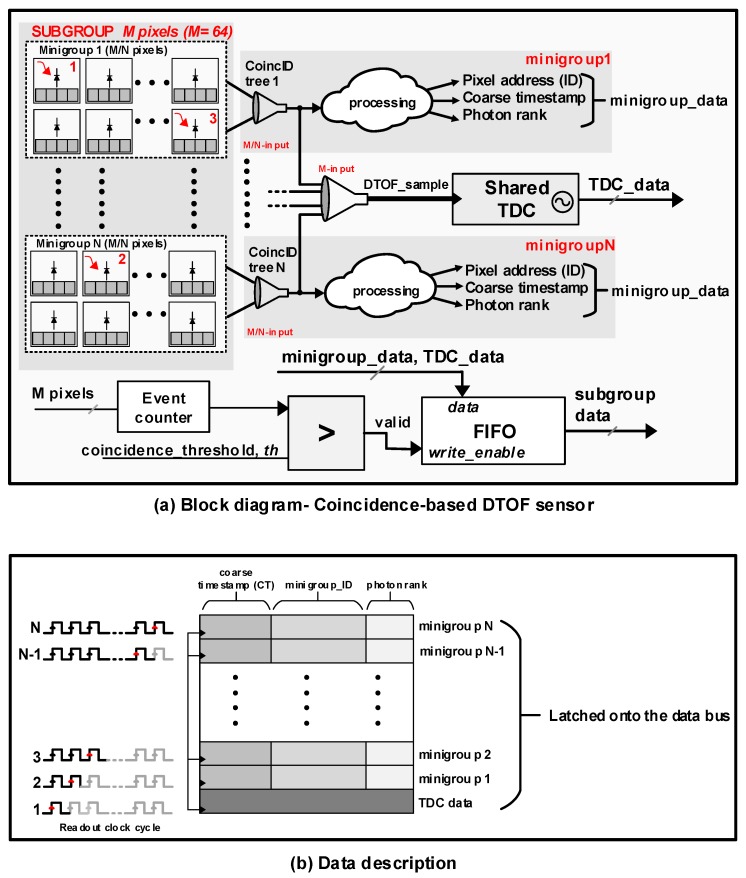
(**a**) A block diagram of a DTOF sensor adapted to detect coincidence and (**b**) description of data from subgroup, that is, minigroups and TDC data.

**Figure 8:**
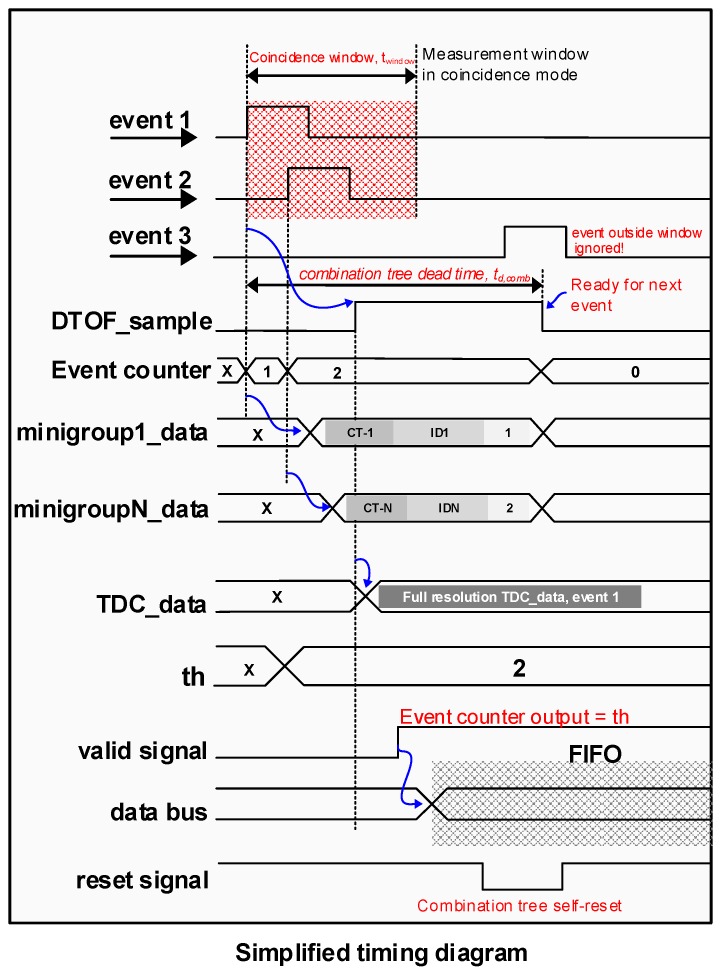
Simplified timing diagram showing the operation of the proposed architecture.

It was seen in Section 2.2 that the DTOF sensor architecture shown in Figure 4 operates predominantly in the negative SBR regime, making it undesirable for a LiDAR application in the presence of high ambient light. In this section, a DTOF sensor model adapted to detect coincidence is proposed. The spatial arrangement alone is similar to the model in Figure 4 while the combination tree itself is significantly modified to enhance SBR not only in a flat uniform target scenario but also addressing the challenge of wide dynamic-range target scenes. Figure 7a shows a high-level block diagram of the proposed DTOF sensor model. Figure 8 shows a timing diagram with the flow of operations by taking an example of three events (marked in red as 1, 2, 3) as shown in Figure 7a.

In the proposed model, the subgroup, sg (*M* pixels), is further clustered into *N* minigroups, mg, comprising of (M/N) number of pixels each. Unlike the approach in Figure 4 where the first incoming event is propagated while blocking/ignoring successive events, the tree in the proposed architecture has a non-blocking nature. Arrival of the first event starts a coincidence window, $t_{window}$ (see event 1 in Figure 8), which can be tunable based on system specifications. While a M-input combination tree propagates the first event (DTOF_sample in Figure 7a and Figure 8) to the TDC, the successive events within the window are preserved and locally processed in the minigroups by the M/N-input combination trees. The combination tree within the minigroups is referred to as the “coincID” tree. Every minigroup, mg, is modeled to provide coarse timestamps with a resolution, $T_{coarse}$, along with generating the binary ID ($log_{2}\left(N\right)$ bit ID) of the pixels contributing events. For *N* number of minigroups, the subgroup can provide data for up to *N* number of detections within the coincidence window as shown in Figure 7b from the data description of the minigroups. As shown in Figure 8, minigroup 1 generates data corresponding to event 1 as a coarse timestamp, CT-1 along with its address referred to as ID1 and the photon rank which indicates the order of the photon in a coincidence window. Similarly, minigroup *N* provides data corresponding to event 2.

There is an event counter in every subgroup which tracks the number of photons within a coincidence window. A comparator logic is used to compare the output of the event counter with a predefined (and variable) coincidence threshold, th. Whenever the event count exceeds th, a signal is considered valid. In the example timing diagram in Figure 8, th=2 and as the event counter output reaches 2, a valid signal is generated. The valid signal enables the data-writing process in the FIFO block following which the data from various minigroups and the TDC data is latched onto a data bus (see data description in Figure 7b). The main purpose of implementing variable thresholds is to address the challenge in wide dynamic range targets where photons from lower reflective parts of the scene need to be captured in the presence of brighter targets. It will be shown in Section 4.2 that there is an evident relationship between the coincidence threshold and the photon activity rate. Additionally, the filtering action through coincidence detection, along with multiple minigroups which enable timestamping of more than one event in a coincidence window also help mitigate pile-up distortion [25] by avoiding unnecessary sampling of the TDC when the threshold condition is not satisfied and processing more than 1 photon through the minigroups.

Typical approaches in TCSPC with coincidence detection include a combination logic which combines the events from multiple pixels on satisfying coincidence but only with a compromise on losing granularity and achievable spatial resolution [11]. When a flash LiDAR is operating in a wide FOV, the sensor may see multiple different targets within the scene, unlike low-FOV systems. Under such scenarios, it is important to capture information from as many targets as possible within the FOV or at least ensure minimal depth errors. The feature of multiple coarse timestamping in the minigroups facilitates this by allowing up to *N* detections within the subgroup by providing the *timestamp* and *ID* data of those *N* detections.

The probability of noise and signal detections are calculated for the proposed architecture in the coincidence mode. Under high background noise conditions, noise events can potentially result in false coincidences as well, where a valid event may be a contribution of either all noise events or a combination of signal and noise events. This is in addition to true coincidences where the contributing events are only due to signal photons. Thus, the probability of the true signal detection will depend on the probability of propagating the false coincidences as well. For a pixel, *i*, the number of noise and signal events per pixel during a coincidence window, $t_{window}$ with a coincidence threshold, th, can be calculated as

(24) $$N_{th,pixel}\left(i\right)=N_{pixel}\left(i\right)\times \left(t_{window}-th\cdot t_{d,spad}\right),$$

(25) $$S_{th,pixel}\left(i\right)=S_{pixel}\left(i\right)\times \left(\left(1/f_{laser}\right)-th\cdot t_{d,spad}\right).$$

**Figure 9:**
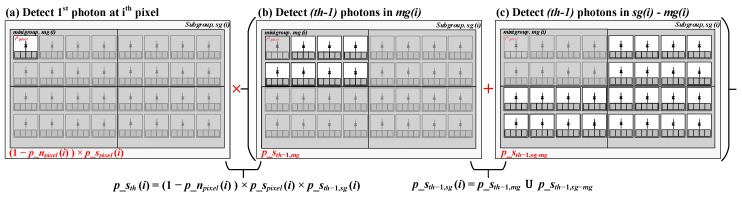
Subgroup, sg(i), demarcated to show various probabilities under coincidence mode to detect (th) number of signal photons.

Figure 9 shows a subgroup example with M=32 pixels with N=4 minigroups containing 8 pixels each, where, regions corresponding to specific probabilities are highlighted for the ease of understanding. The equations on the figure correspond to signal probabilities (Equations (29) and (31)) while the pictorial representation itself is the same for analyzing both, noise and signal.

The probability of detecting a valid noise event (false coincidence) at pixel, *i* (marked as $i^{th}pixel$ in red in Figure 9a), with th number of coincident events in the subgroup, sg(i), is calculated as the conditional probability of detecting a noise event at pixel, *i*, given that (th−1) noise events are detected in rest of the subgroup (Figure 9a).

(26) $$p\_n_{th}\left(i\right)=p\_n_{pixel}\left(i\right)\times p\_n_{th-1,sg}\left(i\right),$$

where $p\_n_{pixel}\left(i\right)$, the probability of detecting 1 event, is calculated as

(27) $$p\_n_{pixel}\left(i\right)=N_{th,pixel}\left(i\right)\exp\left(-N_{th,pixel}\left(i\right)\right).$$

The probability of detecting (th−1) noise photons in rest of the subgroup is calculated from the union operation of individual probabilities of detecting (th−1) noise photons in the minigroup, mg(i) (see Figure 9b) and the rest of the subgroup, sg(i)−mg(i), (see Figure 9c).

(28) $$p\_n_{th-1,sg}\left(i\right)=p\_n_{th-1,mg}\cup p\_n_{th-1,sg-mg}.$$

The effective number of photons in the minigroup, mg(i) and in rest of the subgroup, sg(i)−mg(i), excluding the minigroup to which pixel *i* belongs is calculated from the pixel-wise photon number calculated in Equation (24) and used to calculate $p\_n_{th-1,mg}$ and $p\_n_{th-1,sg-mg}$.

The probability of detecting valid signal events within $t_{window}$ can be calculated as a conditional probability of detecting a signal event in a pixel, *i*, given that no noise photon is detected at *i* and (th−1) signal events are detected in the rest of the subgroup (see Figure 9 showing combined probability).

(29) $$p\_s_{th}\left(i\right)=\left(1-p\_n_{pixel}\left(i\right)\right)\times p\_s_{pixel}\left(i\right)\times p\_s_{th-1,sg}\left(i\right),$$

where $p\_s_{pixel}\left(i\right)$, the probability of detecting 1 signal event, is calculated as

(30) $$p\_s_{pixel}\left(i\right)=S_{th,pixel}\left(i\right)\exp\left(-S_{th,pixel}\left(i\right)\right).$$

Likewise, the probability of detecting (th−1) signal photons in rest of the subgroup is calculated from the union operation of individual probabilities of detecting (th−1) signal photons in the minigroup, mg(i) and the rest of the subgroup, sg(i)−mg(i) (see grouping in Figure 9),

(31) $$p\_s_{th-1,sg}\left(i\right)=p\_s_{th-1,mg}\cup p\_s_{th-1,sg-mg}.$$

Equation (25) is likewise used to compute effective number of photons in the minigroup, $mg_{i}$ and in rest of the subgroup, sg(i)−mg(i) as mentioned for noise photons.

The final conditional probabilities in Equations (26) and (29) for noise ($p\_n_{th}$) and signal ($p\_s_{th}$) events respectively, are used to obtain simulation results described in the next section.

## 4. Simulation Results

All simulations are performed by analytically modeling the sensor architecture on MATLAB using the equations through previous sections.

### 4.1. Single-Point Ranging

**Figure 10:**
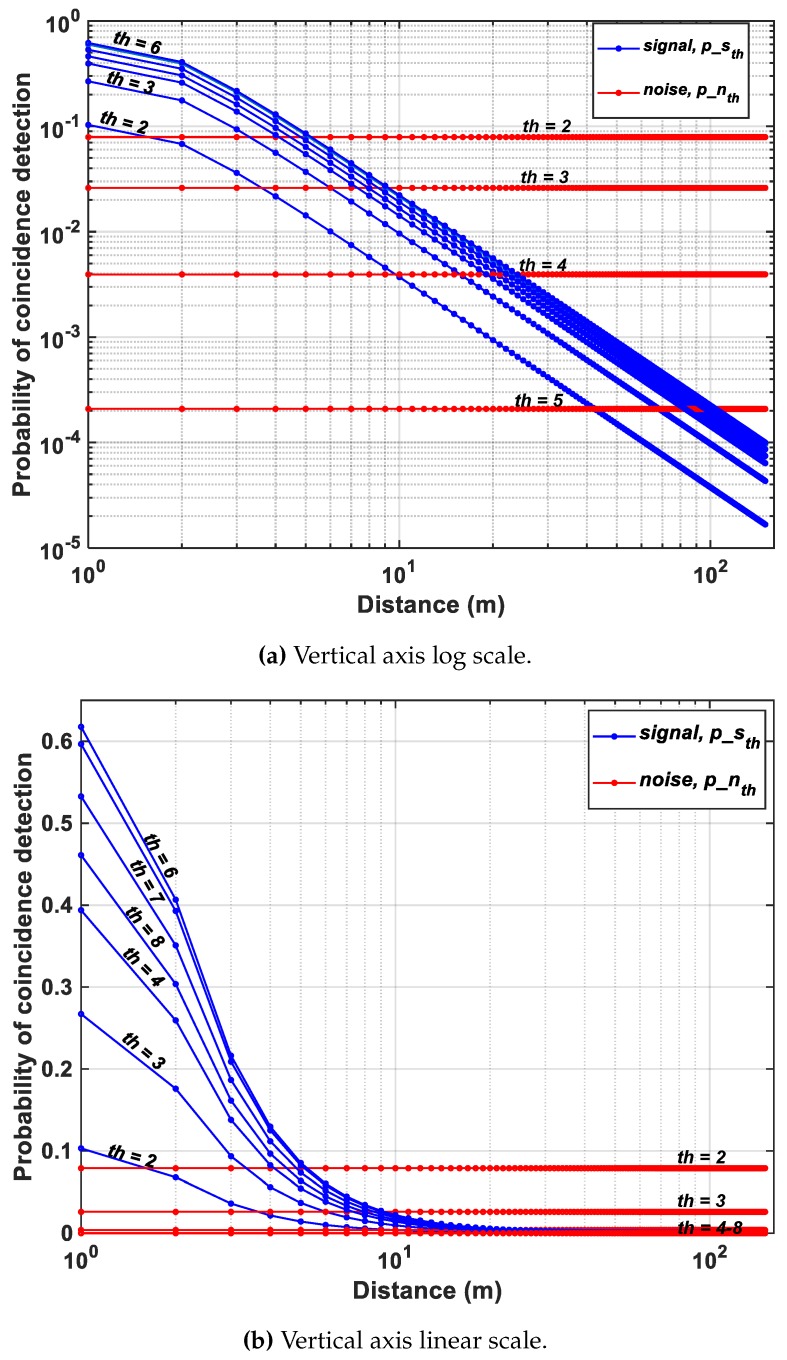
Probability of signal and noise detection at different coincidence thresholds-(**a**) log scale and (**b**) linear scale.

**Figure 11:**
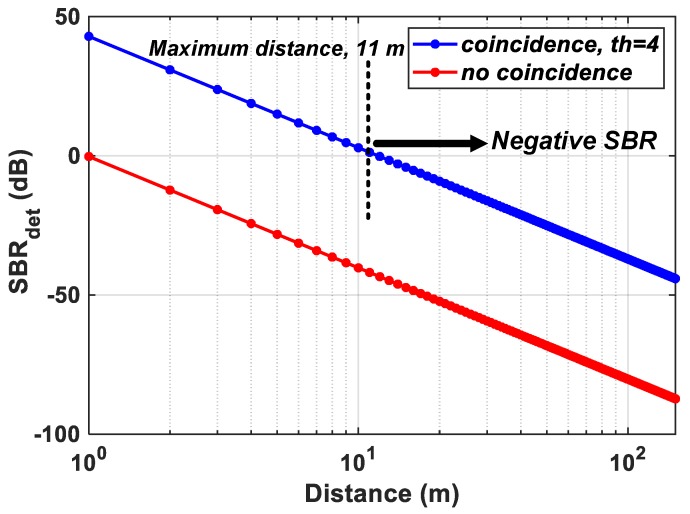
SBR achieved after detection without and with coincidence with th=4.

For the Flash LiDAR scenario described in Section 2.1 and the proposed DTOF model in Section 3, the probabilities of noise and signal detections under coincidence mode are calculated for a flat target at increasing distances, *d*. The subgroup, sg, consists of 64 pixels (8 × 8 ) and the minigroup, mg consists of 4 pixels (2 × 2). Figure 10a,b, show the simulated results on a log and linear scale respectively under a 50 klux background light condition (see Appendix A for results with 100 klux background light for comparison). The probabilities have been plotted for coincidence thresholds, th=2 to th=8 with a $t_{window}\approx 1$ ns. A direct comparison with Figure 5 shows that coincidence evidently increases the probability of signal detection, particularly seen for d=(1−10) m (clearer visibility on linear scale in Figure 10b). While this is true, it can also be seen that for d>10 m, there is significant reduction in signal probability as well. The achieved SBR after detection, $SBR_{det}$ (dB) is plotted for the proposed architecture without and with coincidence, for th=4 in Figure 11. As seen earlier, the sensor operates in a negative SBR regime throughout the unambiguous range under no-coincidence. In addition, it can also be clearly seen that when the sensor is operated under coincidence, for longer ranges, *d*, the returning signal photons are also fewer (inverse square law) and imposing a timing constraint, $t_{window}$, through coincidence does not provide any additional improvement in the SBR. In fact, under the wide-angle FOV flash scenario used in this simulation with 50 klux background noise, the maximum distance is 11 m up to which the the sensor still operates in a positive SBR regime. Beyond this point, the sensor enters the negative SBR region (see marking in Figure 11).

This points to two main corollaries—(1) as concluded in Section 2.2, noise-filtering and thus, SBR improvement provided by optical means such as, bandpass filters, is limited and (2) noise-filtering implemented at the sensor-level also has its limitations. Therefore, the components within a LiDAR system should not be understood as mutually-exclusive in operation. In other words, for a robust operation of a LiDAR, the whole system should be considered as a closed-loop system where there is continuous feedback between the sensor and the rest of the components—illuminator and the optics (indicated in dotted lines in Figure 2). As seen in Figure 10, for longer distances, the fewer number of returning photons can be overcome by a number of parameters, a few of them to name, being,

1. Increasing the outgoing laser power without violating eye-safety regulations
2. Decreasing the FOV, given that at longer distances, a full resolution sensing may not be required
3. At the sensor level, data from multiple pixels may be combined at the expense of lower spatial resolution
4. Time-gating may be another alternative to achieve target-dependent ranging at the sensor level (will be discussed in Section 4.4)

**Figure 12:**
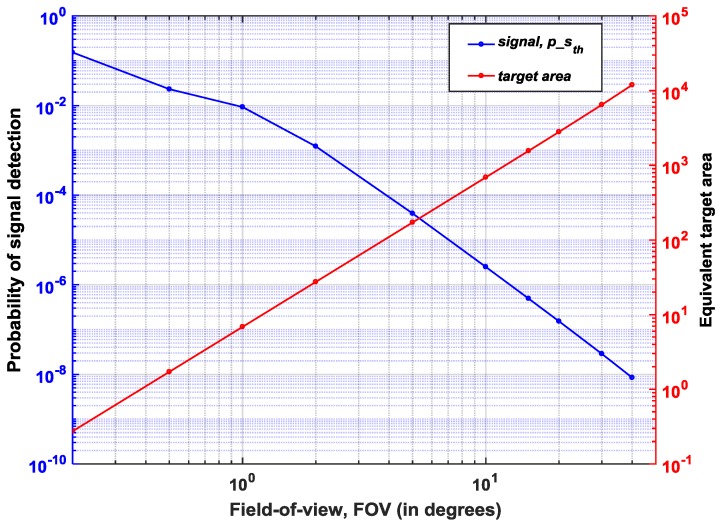
Probability of detection at d=150 m (left vertical axis) and equivalent target area (right vertical axis) at varying FOV (horizontal axis).

Figure 12 shows the simulation of alternative (2) mentioned above to detect signal at 150 m (maximum unambiguous range at $f_{laser}$ =1 MHz) at th=2. The probability of signal detection is plotted with varying FOVs; clearly it can be seen that the success of detection improves with decreasing FOVs as expected and in this example, reaching peak at 0.2${}^{{}^{\circ}}$ FOV. Also, considering a practical scenario, at distances about 50–150 m, it is sufficient to get the range estimation alone and this can be achieved with a very narrow FOV. Figure 12 also shows that a lower FOV and thus, a smaller coverage area result in lower integrated background noise. This naturally improves the SBR and therefore, also the signal detection probability. Certainly, at 150 m, operating with a FOV of 40${}^{{}^{\circ}}$, covers an area of approximately 10,000 m${}^{2}$, which is totally an impractical scenario. Therefore, for longer distances, the illumination unit may be used as a point source, using all the energy of the laser pulse to provide range estimation alone while for shorter distances, the laser energy may be distributed into a wider FOV with an array of points to provide 3D depth map.

### 4.2. 3D Imaging with Wide Dynamic Range Targets

**Figure 13:**
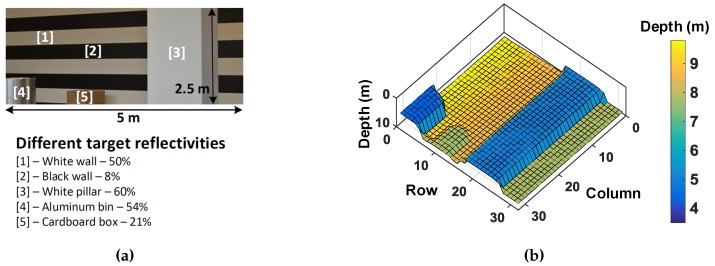
(**a**) Photograph of the example target scene and (**b**) 32 × 32 image reconstructed in a scanning LiDAR setup [8].

The analysis in the previous section showed simulations of single point ranging on a flat target at varying distances. A practical scenario in a flash LiDAR at 40${}^{{}^{\circ}}$ FOV will most likely cover multiple targets within the FOV and these targets may all be different and span over a wide range of target reflectivities. Addressing such scenarios will require a LiDAR with high noise-handling capabilities in a high dynamic range scenario. This section shows simulation results of 3D images reconstructed on a sample scene with wide range of targets. Figure 13a shows a scene where targets range between 8–60% reflectivites. Alongside is a 32 × 32 3D image measured using one of our previous chips [8] in a scanning LiDAR setup in a low-noise environment. This measured image (Figure 13b) is used as an input target scene and fed to the analytical models of the DTOF sensor schemes described in Section 2.2 and Section 3.1 to evaluate the feasibility in a flash LiDAR scenario.

A DTOF sensor with 1024 pixels (32 × 32) is assumed to image the given target scene. The subgroup, sg, consists of 64 pixels (8 × 8) as mentioned previously. The target reflectivities are as indicated in Figure 13a. The probabilities of detection are calculated based on the established equations in Section 2 and Section 3 and using this, the histogram is computed for every pixel. The TOF for every pixel is calculated as the time bin with maximum number of counts in the computed histogram which is then used to reconstruct the combined 32 × 32 depth image. It is assumed that every module (2 × subgroups), in the 32 × 32 sensor array is read out through 8 independent channels (as number of subgroups, $N_{sg}=16$) whenever there is a valid event. The readout clock, $clk_{read}$ is assumed to be around typical digital I/O frequency; 100 MHz is considered for all the simulations herein. The timing throughput per subgroup per second, $t\_counts_{sg}$, can then be calculated as follows,

(32) $$t\_counts_{sg}=\frac{N_{sg}}{2}\times clk_{read}\times N_{mg},$$

where $N_{mg}$ is the number of minigroups.

The histogram statistics is acquired for $t\_counts_{sg}$ number of events for signal and noise. The total number of valid events for noise and signal is referred to as $N_{total}$ and $S_{total}$ respectively. The noise events, $N_{total}$, are modeled as a uniform distribution over the unambiguous range, $1/f_{laser}$ with a bin resolution, $t_{res}$ defined by the total system jitter, $\sigma_{total}$ (Equation (2)) and number of bins, $b=1/f_{laser}/t_{res}$. The signal events, $S_{total}$ are modeled considering the Gaussian nature of the laser pulse and are calculated from the error function of a Gaussian distribution.

**Figure 14:**
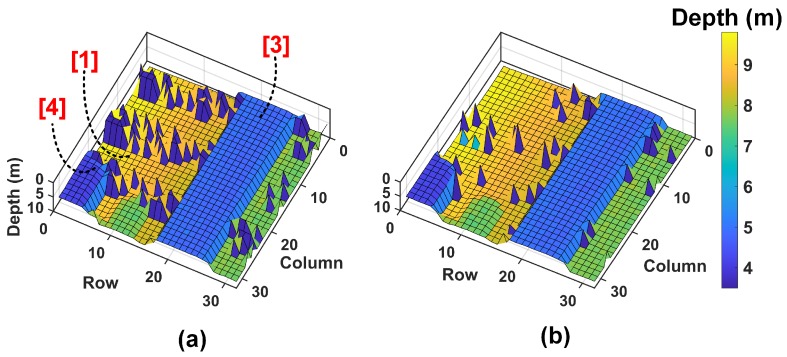
(**a**) Simulated result of the 3D image reconstructed through first-in-win-all scheme and (**b**) coincidence-based proposed architecture.

The first-in-win-all approach, described in Section 2.2 is first simulated. The measured image is fed to this model under a 5 klux background noise condition and in a flash LiDAR setup with $\theta_{H}$ and $\theta_{V}$ are 30${}^{{}^{\circ}}$ and 15${}^{{}^{\circ}}$ respectively. Please note that a 50 klux background noise condition on this simulation showed only noise in the reconstructed 3D image and therefore, a 5 klux noise condition was used to simulate this part alone, to distinguish between noise and signal events and understand the limitations of this scheme. The system timing uncertainty assumed is $FWHM_{total}\approx 530$ ps. The laser parameters remain the same as in previous simulations. Figure 14a shows the obtained 3D image.

Since, first-in-win-all approach described in Section 2.2 does not include any noise filtering mechanism, one can observe the expected degradation in the reconstructed (simulated) image in Figure 14a compared to the measured image in Figure 13b, obtained under minimal noise in a scanning setup.

There is another important observation from this simulation result. It can be seen that the white wall (object (1)), the aluminium bin (object (4)) and the white pillar (object (3)) are readily reconstructed in comparison to the rest of the scene where there is up to 11.9% incorrect sampling. This is also expected of this architecture which has the tendency to propagate events from higher reflective parts (and thus, more number of average events per second) compared to lower reflective parts of the scene.

The coincidence-based DTOF model in Section 3.1 is proposed to mitigate the limitations in a first-in-win-all scheme evident with simulation results from the above plots. The measured image (Figure 13b) is then fed as a target to the proposed model under 5 klux background noise first and simulated. The subgroup, sg, consists of 64 pixels (8 × 8) as mentioned previously and the minigroup, mg (absent in the first-in-win-all scheme) consists of 4 pixels (2 × 2). The reconstructed 3D image from the proposed scheme is shown alongside in Figure 14b for direct comparison with the first-in-win-all (no-coincidence) architecture. As can be seen, coincidence significantly improves the depth measurement with much fewer incorrect samplings.

**Figure 15:**
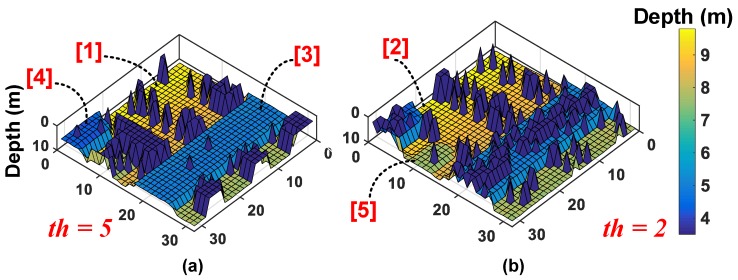
Simulated result of the 3D image reconstructed through proposed DTOF model (**a**) coincidence threshold, th=5 and (**b**) coincidence threshold, th=2.

Following this, the measured image (Figure 13b) is then fed as a target to the proposed model under 50 klux background noise condition and with coincidence. The 3D image results from the simulations are shown in Figure 15, where it can be seen that there is an apparent threshold-selective pattern in the reconstructed 3D image. In Figure 15a, with th=5, it can be seen that objects (1), (3) and (4) being more accurately reconstructed compared to rest of the scene owing to their higher reflectivities, while in Figure 15b, with th=2, objects (2) and (5) take preference over the rest of the scene. This points to an important relationship between coincidence threshold, th and the target reflectivities.

**Figure 16:**
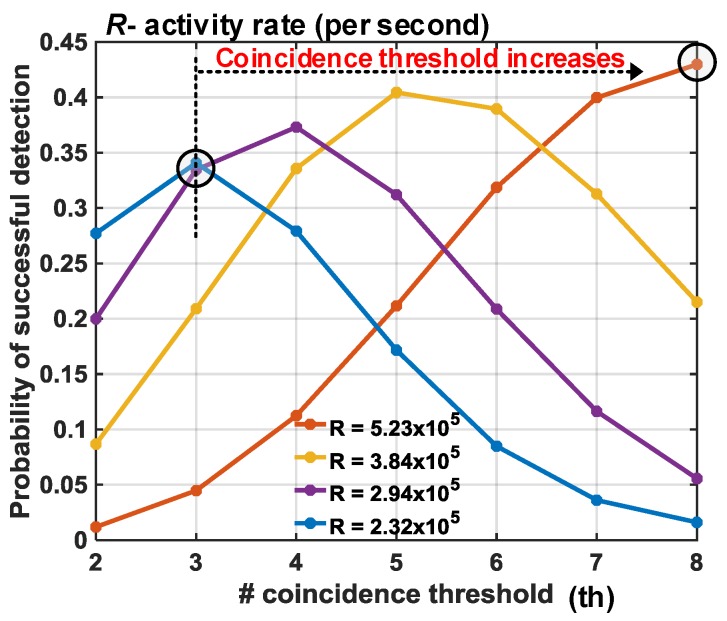
The relationship between coincidence threshold and the incoming activity rate, *R*, received per second.

**Figure 17:**
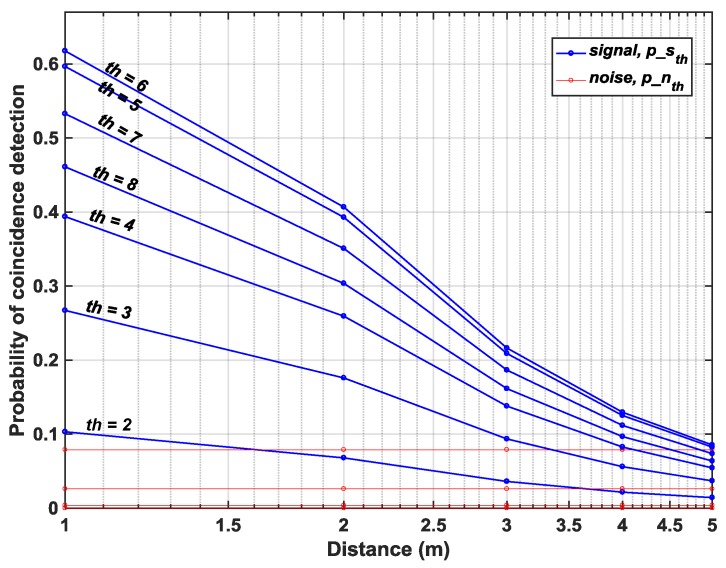
Probability of signal detection in Figure 10b selected to show threshold dependence.

Figure 16 in fact shows how coincidence threshold, th, increases for increasing photon activity, R, to provide a successful detection (temporal error, $\sigma_{total}<230$ ps), implying that a single threshold cannot yield an accurate reconstruction of a scene and a higher (lower) reflective target will imply higher (lower) photon activity rate requiring higher (lower) coincidence thresholds. On revisiting Figure 10b (redrawn for clarity in Figure 17), this relationship is already evident there. For targets at distances, particularly at d=1,2,3 m, where the number of returning photons is higher, the probability of signal detection increases for increasing thresholds, p(d=1,th=6)>p(d=1,th=2) (see Figure 17). However, the probability ceases to rise for thresholds where the number of photons within the coincidence window is lesser than the threshold itself (see p(d=1,th=7),p(d=1,th=8)), given the fact that coincidence detection is performed utilizing photons within the laser pulse.

The threshold, th, marked in Figure 7 can be chosen based on the activity rate of the subgroup to yield maximum success in signal detection. Every subgroup can therefore have a unique threshold and the minigroups can provide data (timing and ID) of the photons within the coincidence window. In fact, the 3D image earlier compared in Figure 14b was simulated with target-specific coincidence threshold in every subgroup over the sensor array.

### 4.3. 3D Imaging and Multiple Timestamping

**Figure 18:**
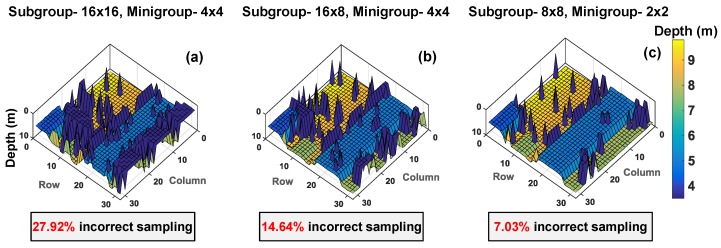
The proposed grouping scheme illustrated for different subgroup and minigroup sizes.

The grouping scheme in the proposed model can be done based on system specifications. For the flash scenario used in this simulation, Figure 18 shows 3D image reconstructions on different subgroup and minigroup sizes where, every subgroup has a unique threshold, th, chosen based on the activity rate (for example, subgroup with white pillar [3], th=5 and cardboard box [5], th=2).

**Figure 19:**
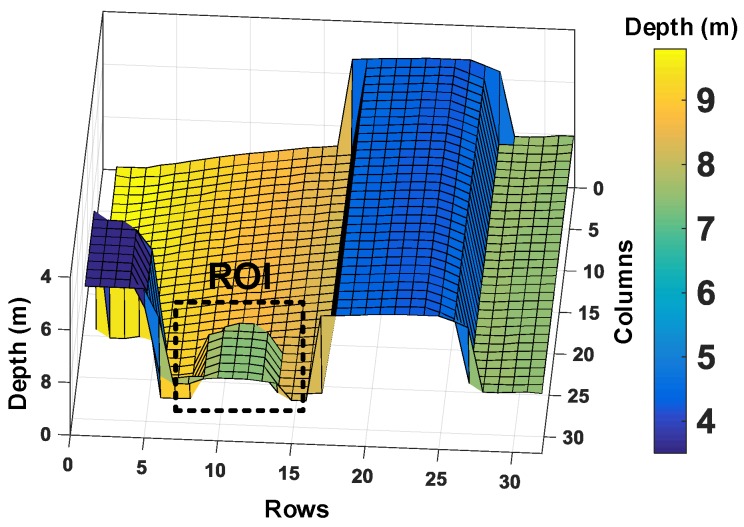
3D image input highlighted with the region of interest (ROI).

As can be seen through Figure 18a–c, a subgroup of 8 × 8 for coincidence and minigroup of 2 × 2 in Figure 18c allows improved 3D reconstruction (only about 7% incorrect sampling) by performing coincidence within a subgroup of 64 pixels (8 × 8). Additionally, the 16 minigroups containing 4 pixels (2 × 2) each enable up to 16 simultaneous TOF measurement within the coincidence window, $t_{window}$. Another objective of enabling multiple timestamping through the minigroups is to reduce the timing uncertainty, which could otherwise be limited to $t_{window}$. It can be understood better by looking at a particular region of interest (ROI) in the example target introduced. Figure 19 highlights the ROI in the target input for this analysis.

**Figure 20:**
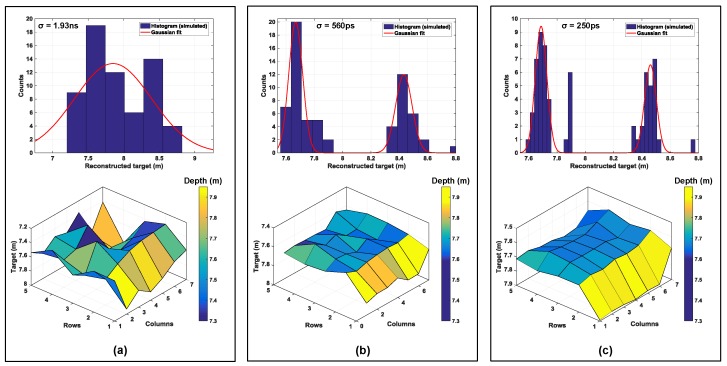
Minigroup timestamping feature (**a**) no timestamping, uncertainty $\approx t_{window}$, (**b**) minigroup timestamping with a resolution, $T_{coarse}\approx 500$ ps and (**c**) minigroup timestamping with a resolution, $T_{coarse}\approx 200$ ps.

Absence of timing information on the contributing events within $t_{window}$ results in an averaging effect in the reconstructed image, with timing uncertainty $\approx t_{window}$. This may not be of concern for very narrow pulses (with small FWHM), however when pulses become wide (on the order of 2–5 ns), this uncertainty can result in depth error estimates. Figure 20a shows this effect where there is no mutliple event timstamping. As can be seen, the reconstructed 3D image has averaged out with respect to the actual target input (see ROI in Figure 19) with an uncertainty, σ=1.93 ns and as seen in the corresponding histogram, the obtained Gaussian fit peaks around the mean value of 7.84 m, deviating from the actual input.

**Figure 21:**
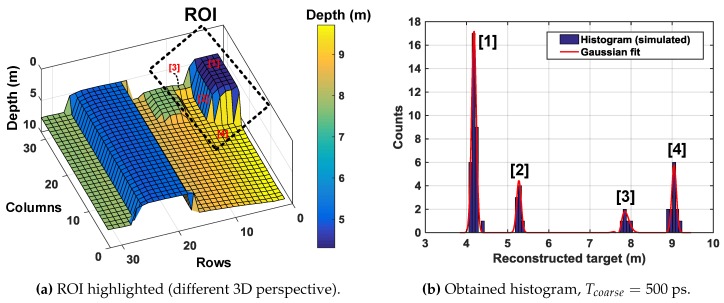
Minigroup timestamping feature for up to four peaks.

Figure 20b,c shows simulations with minigroup timestamping with a resolution of $T_{coarse}$ = 500 ps and $T_{coarse}$ = 200 ps respectively. The histogram in these cases shows two distinct peaks with a bin resolution given by $T_{coarse}$. These peaks are then spatially correlated to the pixels in the minigroup from the IDs generated in each minigroup (as described in Section 3.1). The improvement in timing uncertainty can also be seen in the corresponding 3D images below the histograms. In principle, it is possible to detect up to 16 distinct peaks (number of minigroups = 16) in this example scenario. Figure 21 shows another histogram simulation result on a different ROI, as marked in Figure 21a. As labelled, there are 4 distinct parts in this ROI which are seen as 4 distinguishable peaks on the histogram.

### 4.4. 3D Imaging and Time-Gating

Time-gating is a useful technique to achieve target-selective ranging, particularly useful at long distances. Practical example may include a scene where there may be a retro-reflective object in front of the actual desired target and it may become difficult for the sensor to estimate and reconstruct the actual target situated behind this retro-reflective object due to significant differences in their returning photon rates. Another scenario where time-gating may be effective is in an adverse weather condition due to the presence of scattering medium (such as fog, cloud). Particles in fog/cloud cannot be treated like background noise due to their non-uniform temporal distribution [26,27] which may reflect as distinct peaks in the acquired histogram. Gated imaging can be useful to selectively eliminate unwanted peaks in the light propagation path.

**Figure 22:**
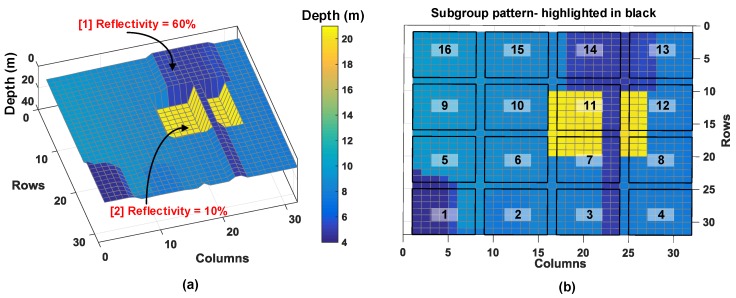
(**a**) Input 3D image used to evaluate time-gating and (**b**) subgroups highlighted with the target scene.

The proposed DTOF sensor is modeled to optionally operate in a time-gating mode to address scenarios such as this. Every subgroup, sg, is modeled to have unique gating windows. A different example target scene, shown in Figure 22a is used as an input to analyze this condition and fed to the proposed DTOF model, now operating under time-gating mode. The number of pixels in the subgroup is assumed to be 8 × 8 with 16 minigroups, each consisting 4 pixels (2 × 2).

Labelled [1] is an object with 60% reflectivity (retro-reflective equivalent) at about 5 m and the desired target, labelled [2], situated at 20 m with 10% reflectivity. This target is reconstructed by the proposed DTOF model with 1024 pixels (32 × 32); the parts of this target as seen by different subgroups is highlighted in Figure 22b for ease of visualization. As numbered above, $sg_{11}$ will be focused for this analysis; it covers a vast portion of the actual desired target (42 pixels) and a small fraction of the retro-reflective equivalent (17 pixels). The proposed DTOF model is simulated without and with gating.

**Figure 23:**
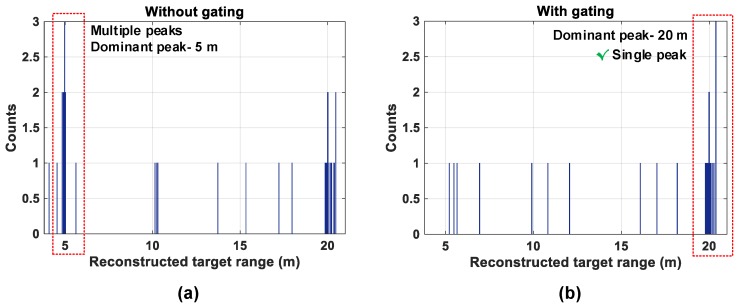
Histogram of $sg_{11}$ (**a**) without gating and (**b**) with gating.

The histogram is then calculated for all the pixels in $sg_{11}$ and the simulation results are shown in Figure 23.

The average photon rate from object (1) is much higher than object (2) and therefore, the subgroup combination tree will propagate more events from object (1) compared to object (2) for a given measurement time, which can potentially mask the desired target photons from a target which may reflect fewer photons (as object (2) in this example). Without gating results in a histogram with two peaks, with a dominant one at 5 m as seen in Figure 23a and another peak at 20 m. In fact, if a desired target was situated at a much longer distance, it may potentially not be possible to recover photons back from this target. Consequently, the presence of a dominant peak from the retro-reflective equivalent in the acquired histogram may mask the presence of the farther target. While the dominant peak from the retro-reflective target is not wrong, the low probability of recovering the farther target peak is of important concern.

Gating will thus, allow us to select and propagate only “desired timestamps” within a gating window, in that way we can mitigate situations as in this example case.

In Figure 23b, the subgroup operates in the gating mode with a gating window of 10 ns around the desired target at TOF≈133 ns (20 m) and the resulting histogram generates only one peak, around the desired target. In general, the sensor can have a moving gate with tunable window lengths to scan through the maximum ambiguous range (≈$c/(2f_{laser})$) to gather an estimate on the desired target and choose the gating window accordingly.

## 5. Conclusions and Future Work

Given that depth sensing involves a number of interdependent challenges, it is imperative to visualize the entire LiDAR system as a closed-loop system wherein, there is a continuous feedback from the sensor to the illumination control logic and/or the optics. Therefore, we proposed an alternative DTOF sensor model in this article, with an architecture suitable for such an implementation. The sensor (or subgroup) is modeled to estimate the returning photon activity from different parts of the scene. This information can then be used to select an appropriate coincidence threshold as seen in Section 4.2, to improve 3D imaging in wide dynamic range scenarios. Passive imaging in the sensor can be used to accumulate incoming photon activity on the sensor for a desirable observation time-window. The output after accumulation can be fed back to a control unit to configure subgroups with appropriate coincidence thresholds. While the feedback and configuration process itself can be very fast in an integrated implementation of the sensor, the major latency will be dictated by the observation window used to accumulate the incoming photon activity.

The feature of subgroup clustering into multiple minigroups enabled with independent timestamping increases the overall timing throughput (up to number of minigroups). This has a direct implication on the histogram processing as well, especially in long distance ranging, by providing multiple depth estimation simultaneously. Additionally, this feature also decreases the timing uncertainty within a detection window (coincidence or gating), the absence of which can otherwise lead to large depth errors, particularly when windows become longer than ≈2–10 ns (≈0.3–1.35 m position error). Multi-pixel timing, counting and ID information as proposed preserves multiple pixel-data even in a shared architecture, which otherwise is typically lost due to the way they are combined [8,11]. Furthermore, the information from multiple pixel events can be used in efficient photon-by-photon processing directly at the hardware-level instead of streaming out the entire raw data for processing off the chip [28].

The analytical model and simulations in this article provide an early validation and aid conceptual understanding of the sensor architecture. The next step will involve transforming this analysis into a SPICE-compatible model to support actual design of the sensor in an integrated circuit. A parallel future work will also involve building ray-tracing models to predict the photon behavior realistically in the light propagation path within the LiDAR system.

## 6. Patents

Direct time-of-flight depth sensor architecture and method for operating of such a sensor (Application PCT/EP2019/066478, 21 June 2019).

## Figures and Tables

**Table 1:** Simulation parameters

Parameter	Value
Average laser power, $P_{avg}$	20 mW
Laser wavelength, $\lambda_{laser}$	780 nm
Repetition rate, $f_{laser}$	1 MHz
Total system FWHM	530 ps
Target reflectivity, *r*	variable, 8–60%
Field-of-view, FOV	15${}^{{}^{\circ}}$–40${}^{{}^{\circ}}$
Background light	variable, 5–100 klux
Sensor resolution	32 × 32
SPAD detector PDP	10%
Pixel fill-factor, FF	50%
Diameter of collecting lens, $D_{lens}$	11 mm
f-number, f#	1.4
focal length, f	15 mm
Lens efficiency, $T_{l}$	0.8
Optical filter passband, $\Delta_{bw}$	20 nm
Filter efficiency, $T_{f}$	0.7

**Figure A1:**
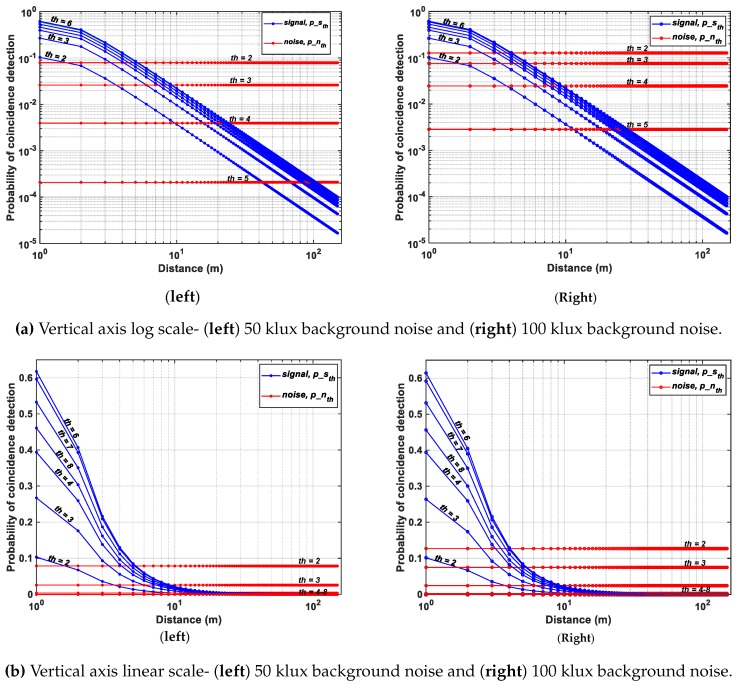
Probability of signal and noise detection at different coincidence thresholds—(**a**) Verticalaxis log scale and (**b**) Vertical axis linear scale.

## Abbreviations

The following abbreviations are used in this manuscript:

TOF	time-of-flight
LiDAR	light detection and ranging
DTOF	direct time-of-flight
ITOF	indirect time-of-flight
SPAD	single-photon avalanche diode
MEMS	micro-electro-mechanical systems
TCSPC	time-correlated single-photon counting
APDs	avalanche photodiodes
SiPM	silicon photomultipliers
TDCs	time-to-digital converters
SBR	signal-to-background noise ratio
FOV	field-of-view
VCSEL	vertical cavity surface-emitting laser
FWHM	full width at half maximum
ROI	region of interest

## Appendix A

The probability of signal and noise detection at different coincidence thresholds under 50 klux and 100 klux background noise condition is plotted below for comparison purposes. The simulation in Figure 10 has been performed in a 32 × 32 array, flash setup, 50 klux background noise and a 10% target reflectivity over a wide angle FOV, 40${}^{{}^{\circ}}$, which already presents a scenario of extremely low SBR condition. The difference between 50 klux and 100 klux results is very low under these simulation conditions as seen below; the probability of noise detection increases slightly in the 100 klux case, while there is not a significant difference in the obtained signal detection probabilities.
